# Linking family planning market census data with consumer experiences in three countries: the Consumer’s Market for Family Planning study protocol and data

**DOI:** 10.12688/gatesopenres.13441.2

**Published:** 2022-05-16

**Authors:** Mark Conlon, Peter Buyungo, Julius Njogu, Anthony Nwala, Susannah Gibbs, Nkemdiri Wheatley, CM4FP Group

**Affiliations:** 1 Population Services International, Washington, DC, 20036, USA; 2 Population Services International Uganda, Kampala, Uganda; 3 Population Services International Global Services Hub, Nairobi, Kenya; 4 Society for Family Health, Abuja, Nigeria; 5 Independent Researcher, Silver Spring, MD, 20902, USA

**Keywords:** Family planning, total market approach, consumer experiences, protocol, open data, Kenya, Nigeria, Uganda

## Abstract

**Background:** The Consumer’s Market for Family Planning (CM4FP) project was designed to address limitations of existing family planning (FP) data sources that prevent a full understanding of the total FP market. CM4FP data provide a picture of the complete supply environment and how consumers experience it.

Study objectives were to 1) test a ring-fenced census approach consisting of an outlet census in a defined geographical area and a household survey in a smaller inner ring, to comprehensively map the total FP market in a local geography; 2) explore FP supply market dynamism through longitudinal data collection from contraceptive outlets; and 3) test a methodology for directly linking household and outlet data to measure the relationship between contraceptive demand and supply.

**Methods: **Data were collected from study sites in Nigeria, Kenya, and Uganda from 2019 to 2020. Longitudinal outlet census data and repeated cross-sectional household survey data from women ages 18-49 were collected at three quarterly time points. Outlets were located in an outer ring geography to encompass locations likely visited by women sampled from a smaller inner ring. Data from women who received a contraceptive method in the past 12 months were linked to data for the outlet from which they received the method.

**Results: **Datasets include product audits for 22,380 individual FP products, collected from a total of 1,836 outlets across 12 study sites. The datasets also contain data from 11,536 female respondents, of whom 1,975 were successfully matched to the outlet where they most recently obtained their method.

**Conclusions: **CM4FP data are available at www.cm4fp.org. This unique dataset enables in-depth exploration of the family planning supply market in addition to interactions between the market and consumer perspectives and behaviors within each study site. The data can also be used to explore novel methodologies to inform future study designs.

## Introduction

Several initiatives in the last decade have foregrounded family planning (FP) as a global public health priority. The FP2020 partnership engaged a variety of global stakeholders—including governments, foundations, civil society organizations, and the private sector—to set a goal of reaching 120 million new contraceptive users in low-income countries by 2020 ( FP2020, 2020). The process for formulating an FP2030 partnership with renewed goals is underway, with the vision of ensuring that all individuals can make their own informed decisions about contraceptive use ( FP2020, 2021). Likewise, the Sustainable Development Goals include a call for universal access to sexual and reproductive health services, including family planning ( UN DESA, 2019).

Alongside these global commitments came a need to monitor progress toward expanding access to contraceptive services. In the process of selecting an overall indicator of progress for FP2020 commitments, several measurement challenges were identified ( Brown *et al.*, 2014). At the time, the primary source of national estimates of contraceptive indicators was Demographic and Health Survey (DHS) data, which are collected approximately every five years ( The DHS Program, 2021a) and are insufficient for timely monitoring of annual progress. Although current modern contraceptive use was selected as the overall metric for monitoring progress, those selecting the metric identified a need for attention to additional aspects of access to contraceptive services, including quality of care ( Brown *et al.*, 2014). Subsequent challenges were to align the goal of increasing contraceptive use, measured from the user’s perspective, to the policy and programmatic resources needed to achieve those goals, and to determine if and how service statistics could be used as valid indicators of progress ( Brown *et al.*, 2014). A need for innovative data approaches was identified to fully understand and address barriers to contraceptive access and monitor progress toward global goals ( Dasgupta *et al.*, 2017).

Existing data sources, as well as those that were developed in the FP2020 era, include certain supply-side information on contraceptive services. The Service Provision Assessment (SPA) survey from the DHS Program gathers supply-side information on the availability of contraceptive methods and services ( The DHS Program, 2021b). Similarly, the Performance Monitoring for Action 2020 (PMA2020) project collects supply- and demand-side data on family planning to track progress toward the FP2020 goals ( Zimmerman *et al.*, 2017). Neither of these data approaches, however, allow for direct, one-to-one linkage of contraceptive users from population-based surveys to the outlets from which they obtained contraceptive services. Although facility-based client exit interviews, such as those included in SPA data, allow for linkage of data from contraceptive users to other facility-level data ( Tessema *et al.*, 2017), these data sources come with limitations of a clinic-based sample as compared to a population-based sample. For example, people who seek services less frequently, such as users of long-acting methods, may be under-sampled. Client exit interviews may also be more vulnerable to social desirability bias associated with being interviewed in a clinical setting. Linkage of facility-level data to population-based survey data could provide key information regarding the relationship between supply-side data and measures of contraceptive use, and on the validity of using service statistics to monitor progress toward global goals.

The Consumer’s Market for Family Planning (CM4FP) project was designed to pilot an innovative approach for directly linking women with the local total market for FP. This was achieved by conducting a census of the FP market in localized geographies and capturing longitudinal data on FP product availability, in tandem with surveys on women nested within the outlet census area, to provide a picture of the complete FP supply environment and how it is experienced by consumers. CM4FP was not designed to be representative beyond its study sites but rather to test a new approach for providing detailed and linked data on FP supply and demand in the same locality, producing in-depth data on total FP markets and women’s contraceptive knowledge, preferences, and contraceptive-seeking behavior within these markets. The CM4FP project was conducted in Nigeria, Kenya, and Uganda from 2019 to 2020, implemented by PSI and its partners: Population Services Kenya (PSK), Society for Family Health (SFH) Nigeria and PSI Uganda. The study aimed to 1) pilot a ring-fenced census approach to comprehensively map the total market for family planning in a local geography; 2) explore dynamism of the FP supply market through longitudinal data collection; and 3) test a new methodology for directly linking household and outlet data to measure the relationship between contraceptive demand and supply. This paper provides an outline of the study methodology and description of the publicly available data.

## Methods

### Study design

The CM4FP project was designed to produce data with detailed information on FP supply and demand in the selected study sites, to deepen understanding of total FP markets and contraceptive knowledge, preferences, and care-seeking within these sites. The study was conducted in Kenya, Nigeria, and Uganda from 2019 to 2020. In each country, four sites were selected from within an urban area of a different size (large, medium, small, and semi-urban). In Uganda, there was a site in a rural area instead of in a semi-urban one. Sites were purposively selected to reflect PSI programmatic interest, donor priorities, a mix of residential and commercial space, and to have crossover with at least one PMA2020 study site in each country (to allow for the potential comparison of CM4FP results with this other, representative survey).

**Figure 1.**
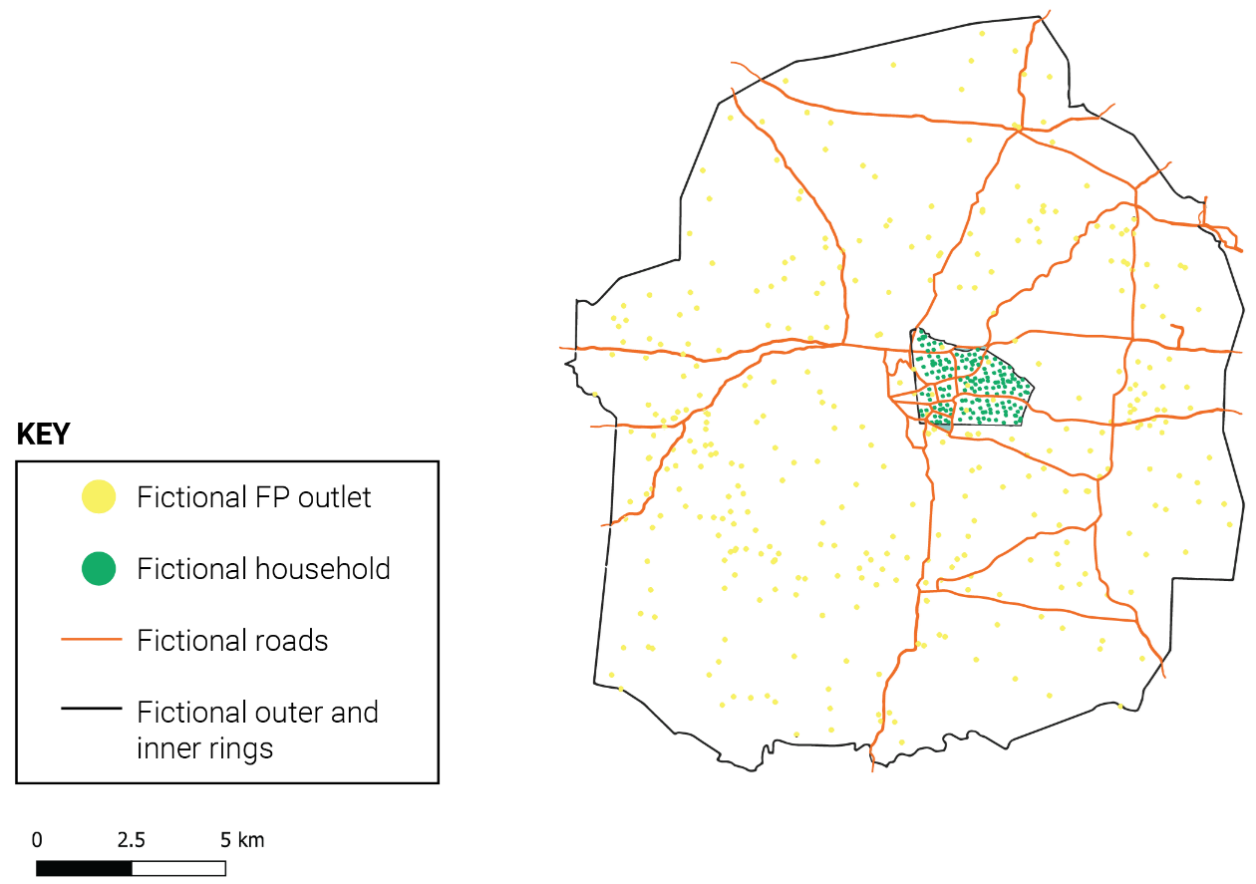
Illustration of outer and inner ring design (hypothetical study site).

Within each study site, data collection was carried out in a single study area consisting of a ring-fenced outer ring and nested inner ring (see Figure 1), to capture the full FP supply environment and directly link women and products/services within the same geography. As a result, the data are not representative of the overall urban areas and should not be interpreted as reflective of the supply or demand landscape at the urban level. Likewise, the purposeful selection of sites means the data are not representative at the regional or national level.

***The ring-fenced design.*** CM4FP used a ring-fenced approach consisting of (1) a census and longitudinal product audit of all outlets and community health workers (CHWs) offering family planning methods or services within a defined geographic outer ring and (2) a repeated cross-sectional household survey of women aged 18–49, sampled from a smaller inner ring centered within the outer ring. The outer ring census aimed to provide a complete picture of the total FP market in the immediate vicinity of surveyed women, who were nested within the inner ring of the study area. This design allowed the study to measure the local total market for family planning as it appears from the respondents’ point of view, and to maximize the chance that surveyed contraceptive users obtained their most recent FP methods from outlets in the outer ring.

***Data collection timeline.*** Data were collected over multiple rounds—longitudinally from outlets and in repeated cross-sections from women. In total, three rounds of quarterly data collection were completed in Kenya and Nigeria, and two in Uganda.

CM4FP data were designed to be collected in multiple rounds over a nine-month period using the following steps: an initial outlet census, four rounds of longitudinal data from the censused outlets in the outer ring, and four rounds of cross-sectional data from women of reproductive age in the inner ring. CM4FP was expanded to include Uganda after the study’s inception, and thus only three rounds of data collection were planned. The outlet audit data, collected quarterly, was designed as a panel to measure changes in the availability of FP products and services. The household data were cross-sectional because of concerns about high loss to follow-up, to avoid biasing respondents across multiple rounds, and because it was unlikely there would be measurable changes in FP use between rounds. The household data were designed to be collected after each round of outlet data, to match with the supply environment as closely as possible. This design also had the practical advantage of allowing the same enumerators in each study site to collect both sets of data.

Due to the COVID-19 outbreak and associated restrictions, data collection was suspended in early 2020 before completion of the final round. As a result, only three of the planned rounds of outlet data were collected in Kenya and Nigeria, while in Uganda we were only able to complete two rounds (see Table 2). Additionally, a second outlet census, planned to accompany the fourth round of outlet data collection in order to measure any changes in the outlet landscape over the life of the project, was also not implemented.

***Measuring the total market for FP.*** The comprehensive mapping and census of outlets within the outer ring areas and FP product and service audits were designed to provide data on the total market for FP in the CM4FP study sites. Geo-coordinates were recoded for outlets. A photo of the exterior (or for CHWs, a brief physical description) was taken, if permitted, to aid direct linking during the household questionnaire.

***Measuring FP supply-side dynamics.*** Outlet data collection was designed as a product audit and gathered information at each visit on each contraceptive method in stock at the outlet on the day of the survey, including brand name, price, any recent stockouts in the previous three months, wholesale/supplier type and cost, and sales volumes. Service readiness indicators were also collected from outlets offering services. For outlets and CHWs offering provider-dependent contraceptive services (e.g., injectable, implants, IUDs), the questionnaire also asked if a woman could receive the service on the day of the interview and if not, the reasons for that limitation.

***Measuring FP demand and use.*** The objective of the household survey was to capture the consumer’s perspectives on the FP market, including identifying where current or recent users of FP obtained their most recent contraceptive method, and to better understand drivers of outlet choice and of FP use more broadly. Data were also collected from FP non-users to ascertain their perceptions and knowledge of the FP supply environment. The survey asked respondents about knowledge of FP methods, current FP use and use history, birth history and fertility preferences. The subset of women who were currently using or had used a modern family planning method in the last 12 months were asked to identify the method source, factors affecting outlet choice, knowledge of the supply market, and experience of stockouts. Household GPS coordinates were recorded for all participating women.

***Directly linking FP users and outlets.*** CM4FP attempted to match users of modern FP methods (current and recent) to the outlet that was the source of their most recent supply of the family planning “survey method” identified during the questionnaire. FP users who obtained this supply themselves (as opposed to someone else, like a partner, providing it) within the past 12 months and within the study area were asked to identify from which outlet or CHW they sourced it. These women were asked to identify the outlet or CHW by name, address or other location information, and/or staff name. No respondent names, including interviewed outlet staff, appear in the final dataset. Enumerators attempted to match this information to a pre-populated list from the CM4FP census. Respondents were also asked to confirm matching search results based on an outlet photo, if available. In a later section of the questionnaire, all women using FP within the past 12 months went through a similar matching process to identify the FP outlet that they reported as being nearest to them.

### Sampling strategy

***Outlet census.*** The geographical boundaries for the outer ring at each site encompassed one or more contiguous wards (Kenya and Nigeria) or parishes (Uganda), that were completely censused to measure the total market for FP products and services within each ring-fenced area. To determine the geographic boundary of the outer rings, an initial target area was selected to capture a total of 600 outlets across all sites in each country. Because of the exploratory nature of the study and the census approach, the number of outlets included in the study was not statistically predetermined. Rather, the target number of outlets was determined pragmatically to allow for a deep dive into localized family planning markets within the constraints of the available budget and timeframe. Fieldwork was concurrent across study sites within each country, and complete wards or parishes were censused and added to the outer ring sequentially until the target sample size for each country was achieved or exceeded. Once the overall target number of outlets for the country had been achieved, the outlet census was completed in the current ward/parish in each study site, and no further wards/parishes were added. The final number of outlets counted in the census varied across sites, largely due to differences in outlet density within wards or parishes ( Table 1); larger urban area outer rings generally had a greater number of outlets in each ward or parish, and small urban, semi-urban, and rural areas had fewer outlets in each ward or parish.

**Table 1.** Sample sizes and geographic areas of outer and inner rings by study site and round.

Site	Number of outlets *	Size of outer ring (km ^2^)	Number of women interviewed	Size of inner ring (km ^2^)
Kenya				
Large urban (Nairobi District)	274	14.3	885	0.2
Medium urban (Nakuru District)	239	35.6	892	3.0
Small urban (Kilifi District)	85	307.3	1,037	1.9
Semi-urban (Migori District)	66	84.3	1,002	13.2
**Total**	**664**		**3,816**	
Nigeria				
Large urban (Lagos State)	159	7.3	862	0.7
Medium urban (Kaduna State)	197	22.3	1,240	2.0
Small urban (Abia State)	178	5.0	1,280	0.6
Semi-urban (Niger State)	138	29.4	1,347	3.0
**Total**	**672**		**4,729**	
Uganda				
Large urban (Kampala District)	167	6.2	756	0.5
Medium urban (Mbarara District)	150	11.8	757	0.5
Small urban (Gulu District)	167	33.3	974	1.0
Rural (Soroti District)	16	54.5	504	21.5
**Total**	**500**		**2,991**	

*Including CHWs

**Table 2.** Dates of data collection by round and study site.

Round	Survey	Kenya	Nigeria	Uganda
1	Outlet	April 30 – May 23, 2019	June 29 – July 17, 2019	October 4 – October 25, 2019
1	Household	July 9 – July 28, 2019	September 21 – October 14, 2021	December 1 – December 22, 2019
2	Outlet	September 7 – September 20, 2019	October 31 – November 14, 2019	January 20 – February 1, 2020
2	Household	September 20 – October 5, 2019	November 22 – December 16, 2019	February 8 – March 22, 2020
3	Outlet	November 27 – December 12, 2019	February 5 – February 18, 2020	N/A
3	Household	January 18 – February 8, 2020	February 26 – March 21, 2020	N/A

***Household surveys.*** The household sample size was calculated to reach a target number of women who had used a modern FP method in the previous 12 months. This target was established to enable exploration of consumer interaction with the localized FP supply market with available time and resources and was not statistically determined. These calculations were informed by the most recent national-level DHS estimates for average household size in urban and rural areas, age distribution, and sex distribution, as well as regional-level DHS estimates of modern contraceptive prevalence rates (mCPR). The target in Kenya and Uganda was 200 women per study site and round. In Nigeria, because of very low levels of modern contraceptive use, the sample size was calculated to reach a target of 125 women who had used a modern method. The geographic boundary of the inner ring was determined by mapping all households in an expanding area (using census enumeration areas in Kenya and CM4FP-produced enumeration areas in Nigeria and Uganda) until the required household sample size was reached. In each survey round, one fourth (in Kenya and Nigeria) or one third (in Uganda) of mapped households from the full listing in each site were sampled randomly from the full household listing. This was done in anticipation of having four (Kenya and Nigeria) and three (Uganda) rounds of data collection, with the intention of inviting all households in each inner ring to participate in the study by the final round. One respondent was randomly chosen from among all eligible household members (women aged 18–49).

### Eligibility criteria

***Outlet census.*** Outlets were eligible for inclusion in the census of FP product and service providers if they had stocked at least one modern FP method (aside from male condoms) or offered any FP services during the past three months. Public and private health provider and health retail outlets of all types within the outer ring, including hospitals, health facilities, pharmacies, patent and proprietary medicine vendors (PPMVs), and drug shops, were screened for inclusion. Outlets that served the military but not the general public were excluded, as were general retailers, bars, hotels, and brothels where only condoms are typically available. In the Lagos and Abia sites in Nigeria, a small number of general retailers/supermarkets offered oral contraceptives and/or emergency contraceptives, so these outlet types were screened and included if eligible. CHWs were also included in the outlet census if they operated in the community as a mobile provider of FP products and not only within brick-and-mortar facilities. Any outlet or CHW from the initial census no longer meeting the inclusion criteria in a subsequent round was excluded from that point forward. All CHWs were excluded from the last round of data collection, as preliminary analysis showed that household survey respondents rarely sourced FP from them (fewer than 0.05% of all women interviewed sourced FP from a CHW in any country).

***Household survey.*** All women aged 18 to 49 living in mapped households within each inner ring were eligible to be selected and invited to participate in the female respondent survey, regardless of current or past use of family planning.

### Ethical approval

Ethical approval was provided by the PSI Research Ethics Board (01.2019 and 04.2019), the AMREF Ethics & Scientific Review Committee in Kenya (P615-2019), the National Health Research Ethics Committee of Nigeria (NHREC/01/01/2007-27/05/2019), the Uganda National Council for Science and Technology review board (SS 5041 and SS 5104), and the Mildmay Uganda Research Ethics Committee (1105-2019). Informed consent was obtained from all household and outlet/CHW survey respondents prior to conducting study procedures. To protect the identify of participants, consent was obtained orally, except in Uganda where consent was written as mandated by the in-country review board.

### Data collection

***Study partners.*** PSI implemented the study in Kenya in partnership with PSK and in Nigeria with SFH. PSI implemented the study in Uganda through its PSI Uganda office. In Kenya and Uganda, enumerators were managed via the research agencies IPSOS and Social Economic Data Center (SEDC), respectively. In Nigeria, enumerators were managed by SFH Nigeria.

***Data collection dates.*** Data were collected during the dates shown in Table 2. On average, data collection lasted 21 days in Round 1, 20 days in Round 2, and 18 days in Round 3. Each outlet survey was scheduled as close as possible to three months following the previous round. The household mapping exercise was conducted between the outlet and household surveys in Round 1. Data collection at the rural site in Soroti District, Uganda, including household mapping, was concurrent with Round 2 in the other Uganda sites.

***Enumerator requirements.*** Data collection was implemented by teams of six to eight enumerators supported by one supervisor and one quality assurance officer per site. 

***Electronic data collection.*** Data were collected electronically on Android-based tablets using Dobility’s open data kit–based software SurveyCTO. Technical support to program the instruments was provided by ikapadata. GPS coordinates were recorded for both outlet and household interviews via SurveyCTO using the tablet hardware, which was supplemented in Nigeria with handheld GPS devices. Photos of outlets, when permitted by the provider, were also taken via SurveyCTO using the tablet camera.

### Data content

The full CM4FP datasets include product audits for 22,380 individual FP products, collected from a total of 1,836 outlets across 12 study sites in Kenya, Nigeria and Uganda. The datasets also contain data from 11,536 female respondents, of whom 1,975 were successfully matched directly to the outlet where they most recently obtained their method. We also provide a further 1.5 million distance/time observations between households and all outlets in surrounding study site outer rings.

***Data standardization and validation.*** During data collection, completed questionnaires were reviewed and corrections were reported by each site’s data collection team. For quality control purposes, backcheck interviews were conducted with 5% of household respondents and outlets, during which outlet characteristics and overall product offerings were confirmed or corrected along with the details of at least two products. Electronic data were reviewed and cleaned by the PSI Washington DC team for quality control purposes. Descriptive outlet information (e.g., type, managing authority) was reviewed by team members in each country to address any identifiable misclassification. Outlet and household GPS coordinates were reviewed for internal consistency by PSI, Washington DC.

***Distance data.*** To protect the identify of participants, coordinates for outlets and household respondents are not included in the publicly available data. However, CM4FP has produced matrices of Euclidian (straight line) distances and modeled travel distances and times between respondent households and all FP outlets within each study site. The modeled travel distances and times assume a least-cost route and speeds for walking, driving, and bicycling. This allows for analysis of distances between households and outlets (rounded to 10 meters), comparison of travel times between households and all outlets in a study site, comparisons of self-report and empirical measures of nearest outlets, and analysis of bypassing behaviors.

***Indicators.*** Select constructed indicators have been precalculated and included with the publicly available data to assist with utilization and interpretation. These indicators are outlined in more detail in the study codebooks and include the following: FP Product and Availability indicators that aggregate product data by method type (e.g., oral contraceptive pills) to denote, for example, whether an outlet offers a given method or had it in stock on the day of the audit; Price and Volume Distributed indicators, provided both in terms of national currency or USD as well as in terms of individual units or as converted to couple years of protection (CYP); Poverty indicators in the household questionnaire, including measures of household poverty using the EquityTool ( Metrics for Management, 2015; Metrics for Management, 2017; Metrics for Management, 2018) and the Poverty Probability Index ( Innovations for Poverty Action, 2012a; Innovations for Poverty Action, 2012b; Innovations for Poverty Action, 2015); Family Planning Use indicators that categorize female respondents’ FP use, based on different combinations of current and previous use and modern/traditional methods; and Matching Process indicators that summarize respondents’ status with respect to the matching process, such as eligibility to attempt matching and which types of outlet information were used to match respondents successfully.

***Accessing data.*** Fully anonymized CM4FP household and outlet data, and distance data between households and outlets are publicly available by request at www.cm4fp.org.

### Use of data

The CM4FP data enable in-depth exploration of the localized FP supply market, in addition to interactions between the FP market and consumer perceptions and behaviors within each study site. The data can also be used to explore the potential use cases for the study’s novel design features to inform the design of future studies with similar aims across disciplines. Illustrative examples of uses for the data include the following:

- In-depth exploration of the total FP market in selected study sites:
  - Assess availability and extent of FP services and commodities delivered outside of the public health system, including private providers and informal outlets such as drug shops and pharmacies
  - Explore FP supply microdata, including audits of FP commodities that assess branding, price, stockouts, and other characteristics
  - Describe FP product and service availability dynamics (such as stockouts or pricing) within individual outlets
- Comparison of the observed FP market with the contraceptive knowledge, attitudes, and behaviors of local female residents:
  - Examine concordance between women’s perceptions of the overall FP market such as availability, cost, or proximity compared to the observed FP supply
  - Unpack women’s preferences and decision-making in FP outlet and method selection
  - Explore why and to what extent women seek FP care and products at outlets that are not the most geographically proximate
- Testing of methodologies for assessing supply markets and consumer interactions (for further application in FP or across disciplines):
  - Assess the validity, feasibility, and efficiency of various sampling approaches to estimate local consumer product markets, such as via simulated sampling of the census data
  - Evaluate direct and indirect methods for linking individual respondents to the local consumer product supply environment

CM4FP has produced a Future Analysis Guide containing specific research questions identified by the study team that may be of interest for future research. This guide is available online at www.cm4fp.org alongside the CM4FP data and other study materials.

### Limitations

CM4FP aims to spark dialogue about the opportunities and challenges associated with the piloted methodologies, and advance future research approaches for the measurement of FP indicators. As with all studies, CM4FP has limitations that are important to consider, for both analysis of the CM4FP dataset and for future applications of similar methodologies. First, the study was not designed to provide representative estimates at the national, regional, or municipal level, nor is it representative of the overall urban areas in which its sites were located, but rather provides in-depth localized FP market data. While the localized nature of the data does not necessarily preclude aggregation or comparison of data across sites, it will be most appropriate to conduct analyses within each site individually and to qualitatively interpret any similarities or differences between sites.

Second, the study’s outlet census design planned to identify outlets within the outer ring in the first and the final rounds via complete mapping, to identify changes in the supply landscape over the study period. In intermediate rounds, identified outlets were to be revisited each quarter (including if not previously reached), but no new outlets were to be identified or visited. The cessation of field activities due to the COVID-19 pandemic, however, resulted in a number of disruptions. The study’s final round of data collection was not conducted, meaning there were three rounds completed in Kenya and Nigeria and two rounds in Uganda. Furthermore, missing the final round meant that CM4FP was not able to identify additions to the outlet landscape (e.g., outlets that had opened or begun offering FP). This activity would have provided useful additional insights on changes to the supply landscape over a nine-month period, and the extent to which the comprehensiveness of quarterly and annual panel data was affected by market entries in the study areas, among others.

Third, only a limited proportion of respondents were eligible for direct linking to their most recent source of FP. All women aged 18–49 from selected households were eligible for the female survey, and inclusion was not based on whether they were current/recent FP users. This approach enables analysis of perceptions and level of knowledge about the FP supply environment from non-users, a feature not offered by studies that link women to source outlets through client exit interviewing or client follow-up. Piloting this approach provided useful insights on the feasibility and value of linking women sampled based on their household residence to the source of their contraceptive supply. However, the number of respondents that we could attempt to directly link to an outlet was limited to 1) current or recent users and 2) those in this subgroup that had personally obtained a contraceptive method from an outlet within the study area and within the twelve-month recall period. As a result, a small proportion (ranging from 7% to 36%) of the female respondents in each site could be directly linked to their source outlet. While the CM4FP study design improved upon prior analytic approaches that rely on the assumption that women seek services at the nearest outlet, which may not be the case ( Elewonibi *et al.*, 2020), some women obtained methods from outlets beyond the outer ring study area.

The relatively small number of women who were directly linked may preclude sub-population analysis (e.g., users of a specific FP method and/or examine multiple demographic characteristics) in the linked sample. CM4FP shows the feasibility of direct linking, but future application of approaches to directly link FP users with their supply should consider the specific needs for data from non-users, alongside the resources needed for initially sampling the large numbers of women (and users) who do not then go on to form part of the final linked sample.

## Data availability

All CM4FP data, related documentation and questionnaires are available at www.cm4fp.org. Any researcher wishing to access the data is required to complete an application form on the CM4FP website, which requires the creation of a Harvard Dataverse account prior to requesting data access. Any questions regarding applications for data access may be addressed to research@psi.org.
