# Supplementary material for: Linking family planning market census data with consumer experiences in three countries: the Consumer’s Market for Family Planning study protocol and data
Source: Gates Open Res. 2022 May 16;5:176. Originally published 2021 Dec 8. [Version 2] doi: 10.12688/gatesopenres.13441.2 (PMC9114377; doi:10.12688/gatesopenres.13441.2)
Supplement: Peer review history

# Peer review: Reviewer response for version 2

**DOI:** 10.21956/gatesopenres.14934.r32076

**Published:** 2022-05-17

**Authors:** Philip Anglewicz

**Affiliations:** 1 Department of Population, Family and Reproductive Health, Johns Hopkins Bloomberg School of Public Health, Baltimore, MD, USA

I have no further comments. The authors have addressed my previous suggestions, and I congratulate them for their important contribution to the field.

Are sufficient details of methods and materials provided to allow replication by others?

Partly

Is the rationale for creating the dataset(s) clearly described?

Yes

Are the datasets clearly presented in a useable and accessible format?

Yes

Are the protocols appropriate and is the work technically sound?

Partly

Reviewer Expertise:

Demography, family planning, contraceptive dynamics, survey and research methods, longitudinal panel study designs.

I confirm that I have read this submission and believe that I have an appropriate level of expertise to confirm that it is of an acceptable scientific standard.

# Peer review: Reviewer response for version 1

**DOI:** 10.21956/gatesopenres.14698.r31882

**Published:** 2022-04-04

**Authors:** Jennifer Winston

**Affiliations:** 1 Carolina Population Center, University of North Carolina at Chapel Hill, Chapel Hill, NC, USA

The article provides a detailed summary of the data design and collection process for the Consumer's Market for Family Planning project. The data are a unique resource to allow for geographic and other analyses of the family planning supply environment in Kenya, Nigeria, and Uganda.

 While there is extensive information provided about the ring-fenced design, I would have liked to have seen additional information about how each sampling area was selected. 

 In addition, the article notes that 1,975 of 11,536 female respondents were successfully matched to their FP supplier. It would be helpful to know how many of the 11,536 respondents were FP users. Among FP users, it would be helpful to know the reasons why FP users were unsuccesfully matched to their suppliers.

Are sufficient details of methods and materials provided to allow replication by others?

Yes

Is the rationale for creating the dataset(s) clearly described?

Yes

Are the datasets clearly presented in a useable and accessible format?

Yes

Are the protocols appropriate and is the work technically sound?

Yes

Reviewer Expertise:

My research focuses on family planning and maternal and child health.  I am trained as a medical geographer, and whenever possible, I try to incorporate spatial considerations into my research.

I confirm that I have read this submission and believe that I have an appropriate level of expertise to confirm that it is of an acceptable scientific standard.

# Peer review: Reviewer response for version 1

**DOI:** 10.21956/gatesopenres.14698.r31566

**Published:** 2022-01-07

**Authors:** Philip Anglewicz

**Affiliations:** 1 Department of Population, Family and Reproductive Health, Johns Hopkins Bloomberg School of Public Health, Baltimore, MD, USA

This study is a valuable effort to examine supply and demand dynamics of contraceptive use, by using an innovative study design of concentric circles; households were sampled in the inner circle and health facilities providing contraceptive methods were sampled in the larger circle. Since many population-based datasets do not permit one to link users with the facility from which they received their service, this is an interesting and valuable approach. Overall, this is a compelling approach and I’m interested to see the main results.

 A few questions and comments:

 The manuscript includes a description of two data courses for supply-side information on contraceptive method and service availability, focusing on PMA and DHS data. However, another approach to linking clients with facilities is through client exit interviews linked to facility surveys (and there are drawbacks to this approach), so the study might also want to describe and discuss this approach. 

 A critical assumption of the ring-fence study design is that women tend to visit health facilities that are within/around their neighborhoods. If they don’t, then the immediate FP facility environment doesn’t really matter for their contraceptive use. Instead, women could visit facilities closer to where they work (and they may not work close to where they live), and/or rural women may travel to cities for health care visits. What do we know from the literature about how women select facilities to visit for services and how far they tend to travel? It would be useful to include this as background justification for the study. 

 Since this study is designed to be innovative, it's not necessarily problematic that the sites aren’t representative of the urban or rural area, but it would still be useful to know how the exact sites were selected. 

 It appears that the size of the outer ring was not determined by distance from the inner ring, but by the size needed to reach 600 facilities across all sites within the country - is this correct? If so, how was this number determined and deemed to be sufficient? How did the study decide the number of facilities in each of the several sites within the country? Overall, it’s not clear whether the study based their sample size of facilities on achieving 600 total or selecting a predetermined number of wards or parishes and selecting all facilities that lied within.

 The study states that a “small proportion (ranging from 7% to 36%) of the female respondents in each site could be directly linked to their source outlet”, and that others couldn’t be linked because they were not using contraception or didn’t visit a facility within the study area - what were the proportions of each for the study sites?

Are sufficient details of methods and materials provided to allow replication by others?

Partly

Is the rationale for creating the dataset(s) clearly described?

Yes

Are the datasets clearly presented in a useable and accessible format?

Yes

Are the protocols appropriate and is the work technically sound?

Partly

Reviewer Expertise:

Demography, family planning, contraceptive dynamics, survey and research methods, longitudinal panel study designs.

I confirm that I have read this submission and believe that I have an appropriate level of expertise to confirm that it is of an acceptable scientific standard, however I have significant reservations, as outlined above.

# Response

**Published:** 2022-05-11

**Authors:** Susannah Gibbs

**Affiliations:** Population Services International, USA

**We thank our reviewers for their extremely helpful and thoughtful comments and questions on this manuscript, and we have responded below.**

**Reviewer: Philip Anglewicz**

 This study is a valuable effort to examine supply and demand dynamics of contraceptive use, by using an innovative study design of concentric circles; households were sampled in the inner circle and health facilities providing contraceptive methods were sampled in the larger circle. Since many population-based datasets do not permit one to link users with the facility from which they received their service, this is an interesting and valuable approach. Overall, this is a compelling approach and I’m interested to see the main results.

 A few questions and comments:

 The manuscript includes a description of two data courses for supply-side information on contraceptive method and service availability, focusing on PMA and DHS data. However, another approach to linking clients with facilities is through client exit interviews linked to facility surveys (and there are drawbacks to this approach), so the study might also want to describe and discuss this approach. 

***Response: **We have expanded the background information to include discussion of client exit interviews as suggested:*

*“Neither of these data approaches, however, allow for direct, one-to-one linkage of contraceptive users from population-based surveys to the outlets from which they obtained contraceptive services. Although facility-based client exit interviews, such as those included in SPA data, allow for linkage of data from contraceptive users to other facility-level data (Tessema et al., 2017), these data sources come with limitations of a clinic-based sample as compared to a population-based sample. For example, people who seek services less frequently, such as users of long-acting methods, may be under-sampled. Client exit interviews may also be more vulnerable to social desirability bias associated with being interviewed in a clinical setting. Linkage of facility-level data to population-based survey data could provide key information regarding the relationship between supply-side data and measures of contraceptive use, and on the validity of using service statistics to monitor progress toward global goals.”*

 A critical assumption of the ring-fence study design is that women tend to visit health facilities that are within/around their neighborhoods. If they don’t, then the immediate FP facility environment doesn’t really matter for their contraceptive use. Instead, women could visit facilities closer to where they work (and they may not work close to where they live), and/or rural women may travel to cities for health care visits. What do we know from the literature about how women select facilities to visit for services and how far they tend to travel? It would be useful to include this as background justification for the study. 

***Response: **We have added test on bypassing the nearest facility for contraceptive services and on visiting facilities outside the study area within our discussion of the study limitations:*

*“While the CM4FP study design improved upon prior analytic approaches that rely on the assumption that women seek services at the nearest outlet, which may not be the case (Elewonibi et al., 2020), some women obtained methods from outlets beyond the outer ring study area”*

 Since this study is designed to be innovative, it's not necessarily problematic that the sites aren’t representative of the urban or rural area, but it would still be useful to know how the exact sites were selected. 

***Response: **The sites were selected purposively to represent a range of geographies (large urban, medium urban, small urban, and semi-urban/rural) for pilot testing the methodology. Sites were selected in discussion with in-country teams and the donor, who suggested the project  have at least one site in common with the PMA2020 project to allow for potential comparison of results. As noted in the manuscript, data from these sites should not be interpreted as representative of larger geographies or of the country as a whole.*

 It appears that the size of the outer ring was not determined by distance from the inner ring, but by the size needed to reach 600 facilities across all sites within the country - is this correct? If so, how was this number determined and deemed to be sufficient? How did the study decide the number of facilities in each of the several sites within the country? Overall, it’s not clear whether the study based their sample size of facilities on achieving 600 total or selecting a predetermined number of wards or parishes and selecting all facilities that lied within.

***Response: **We have made edits to the manuscript text to clarify that the words or parishes were censused in their entirety concurrently across study sites within each country until the target sample size for the country (n = 600) was achieved or exceeded: “Fieldwork was concurrent across study sites within each country, and complete wards or parishes were censused and added to the outer ring sequentially until the target sample size for each country was achieved or exceeded. Once the overall target number of outlets for the country had been achieved, the outlet census was completed in the current ward/parish in each study site, and no further wards/parishes were added.”*

*We have also added information on how the target number of 600 outlets was determined: “Because of the exploratory nature of the study and the census approach, the number of outlets included in the study was not statistically predetermined. Rather, the target number of outlets was determined pragmatically to allow for a deep dive into localized family planning markets within the constraints of the available budget and timeframe.”*

 The study states that a “small proportion (ranging from 7% to 36%) of the female respondents in each site could be directly linked to their source outlet”, and that others couldn’t be linked because they were not using contraception or didn’t visit a facility within the study area - what were the proportions of each for the study sites?

***Response:** Analyses of results from the linkage process are presented in a separate manuscript that is in progress and will be published soon. That publication will include details of the proportions of participants who were not linked because they were not contraceptive users or because they did not visit a facility within the study area.*
